# *Parasabella* Bush, 1905, replacement name for the polychaete genus *Demonax* Kinberg, 1867 (Annelida, Polychaeta, Sabellidae)

**DOI:** 10.3897/zookeys.60.547

**Published:** 2010-10-07

**Authors:** María Ana Tovar-Hernández, Leslie H. Harris

**Affiliations:** 1 Departamento de Ecología Acuática, El Colegio de la Frontera Sur, Unidad Chetumal, Avenida Centenario km 5.5, 77900, Chetumal, Quintana Roo, México; 2 Geomare A.C. Av. Miguel Alemán 616-4B, Col. Lázaro Cárdenas, 82040, Mazatlán, Sinaloa, México; 3 Natural History Museum of Los Angeles County, 900 Exposition Boulevard, Los Angeles, CA, 90007

**Keywords:** Demonax, Parasabella, Sabellidae, fan worms

## Abstract

Parasabella Bush, 1905 is reintroduced as a replacement name for Demonax Kinberg, 1867 (Annelida: Polychaeta: Sabellidae) which is a junior homonym of Demonax Thomson, 1860 (Insecta: Coleoptera: Cerambycidae).

## Introduction

Kinberg (1867) established the new genus Demonax for four new species of sabellid polychaetes that were found in Honolulu (Hawaii), San Lorenzo (Perú) and Valparaíso (Chile). This name is a junior homonym of Demonax Thomson, 1860, a genus of round necked longhorn beetles (Insecta: Coleoptera: Cerambycidae). The coleopteran name is well entrenched in the literature (Dauber 2006, 2008, Guo and Chen 2005); 60 references found in Zoological Record on 14 June 2010; and recognized in Nomenclator Zoologicus). According to the Article 60 of the ICZN (1999), a junior homonym must be rejected and replaced either by an available and potentially valid synonym (Art. 23.3.5) or, for lack of such name, by a new substitute name. Parasabella Bush, 1905 is the oldest available name among the synonyms of Demonax Kinberg and here is reintroduced for the replacement of Demonax Kinberg, 1867.

## Systematics

### Parasabella

Bush, 1905 reestablished

Demonax Kinberg 1867: 354 (not Thomson 1860); 1910: 72.– Johansson 1925: 26–27; 1927: 136.– Knight-Jones 1983: 254.– Perkins 1984: 292–293.– Knight-Jones and Walker 1985: 605.– Fitzhugh 1989: 75–76.– Giangrande 1994: 229–230.
Parasabella Bush 1905: 191, 199–200.– Johansson 1927: 136.
Distylidia Hartman 1961: 129.– Fauchald 1977: 138.– Banse 1979: 870.

#### Type species:

Demonax krusensterni, subsequent designation by Bush (1905).

#### Remarks:

Kinberg (1867) described the new genus Demonax for four new species: Demonax krusensterni and Demonax cooki from Honolulu, Demonax leucaspis from San Lorenzo and Demonax incertus from Valparaíso, and Demonax tilosaulus (Schmarda, 1861) also from Valparaíso. The specimen reported by Kinberg as Demonax tilosaulus (not Sabella tilosaula Schmarda, 1861) is a Chone species according to Hartman (1959: 514). As Perkins (1984) noted, designation of Demonax krusensterni as the type-species by Bush (1905) was unfortunate since figures of the other three new species were published posthumously in the second part of Kinberg’s paper on the polychaetes of the Eugenie Expedition (Kinberg 1910) and the holotype of Demonax krusensterni is in poor condition (Johansson 1925, Perkins 1984).

Johansson (1925) reexamined Kinberg’s Demonax types and commented that Demonax leucaspis, Demonax incertus,and Demonax cooki and questionable Demonax krusensterni were all exemplars of a single species. In 1927, Johansson included these species in synonymy under Demonax leucaspis, with Demonax krusensterni as a questionable synonym. Hartman (1959), following Johansson (1927), designated Demonax leucaspis as the type-species, a mistake that violates Article 69 of the ICZN (1999). Fauchald (1977) and Banse (1979) followed Hartman (1959) while Knight-Jones (1983), Perkins (1984), Fitzhugh (1989), Giangrande (1994) and Gambi et al. (2001) considered Demonax krusensterni as the type-species of Demonax. Knight-Jones (1983) suggested that the syntypes of Demonax cooki could well be juvenile specimens of Demonax krusensterni showing regeneration after damage. Perkins (1984) included Demonax incertus, Demonax cooki and questionably Demonax krusensterni under the name Demonax leucaspis.

Parasabella is currently represented by the following 25 species, 24 of which are new combinations:

Parasabella aberrans (Augener, 1926), comb. n.

Type locality: New Zealand.

Parasabella albicans (Johansson, 1922), comb. n.

Type locality: Japan.

Parasabella aulaconota (Marenzeller, 1884), comb. n.

Type locality: Japan.

Parasabella brevithoracica (Pillai, 1961), comb. n.

Type locality: Nachikuda, Ceylon.

Parasabella cambrensis (Knight-Jones & Walker, 1985), comb. n.

Type locality: Liverpool Bay, UK.

Parasabella columbi (Kinberg, 1867), comb. n.

Type locality: La Plata, Argentina.

Parasabella fernandezensis (Augener, 1922), comb. n.

Type locality: Juan Fernandez Island, Chile.

Parasabella flecata (Hoagland, 1919), comb. n.

Type locality: Puerto Rico.

Parasabella jamaicensis (Augener, 1924), comb. n.

Type locality: Kingston, Jamaica.

Parasabella japonica (Moore & Bush, 1904), comb. n.

Type locality: Japan.

Parasabella krusensterni (Kinberg, 1867), comb. n.

Type locality: Honolulu, Hawaii.

Parasabella lacunosa (Perkins, 1984), comb. n.

Type locality: Hutchinson Island, Florida.

Parasabella langerhansi (Knight-Jones, 1983), comb. n.

Type locality: Madeira.

Parasabella leucaspis (Kinberg, 1867), comb. n.

Type locality: San Lorenzo, Chile.

Parasabella media Bush, 1905.

Type locality: Alaska.

Parasabella microphthalma (Verrill, 1873), comb. n.

Type locality: Vineyard Sound, Massachusetts.

Parasabella oculea (Pillai, 1965), comb. n.

Type locality: Manila Bay, Philippines.

Parasabella pallida (Moore, 1923), comb. n.

Type locality: Santa Cruz, California.

Parasabella polarsterni (Gambi, Patti, Micaletto & Giangrande, 2001), comb. n.

Type locality: Weddell Sea.

Parasabella rufovittata (Grube, 1881), comb. n.

Type locality: Singapore.

Parasabella rugosa (Moore, 1904), comb. n.

Type locality: San Diego, California.

Parasabella saxicola (Grube, 1861), comb. n.

Type locality: Chero, Adriatic Sea, see note below.

Parasabella tenuicollaris (Grube, 1870), comb. n.

Type locality: Adriatic Sea.

Parasabella tommasi (Giangrande, 1994), comb. n.

Type locality: Brindisi, Adriatic Sea.

Parasabella torulis (Knight-Jones & Walker, 1985), comb. n.

Type locality: Liverpool Bay, UK.

## Discussion

P. Knight-Jones considered two “types” of Sabella saxicola. One (ZMB Q 5198) agrees with Grube’s description and his further comment (1870) emphasizing that Sabella saxicola lacked radiolar eyes. This was re-described and illustrated by Knight-Jones (1983) as Demonax saxicola. Later, Knight-Jones et al. (1991: 850) suggested the synonym of Demonax saxicola (Grube, 1861) and Demonax brachyona (Claparède, 1870) (as brachychona, spelling variation), wrongly preferring the use of Claparède’s name. This was later followed by Giangrande (1994: 231). According to the ICZN (1999), Statement of the Principle of Priority (23.3.5), the name *saxicola* has priority over *brachyona*.

The other specimen considered as “type” by P. Knight-Jones (MPW 372) cannot be regarded as a syntype because it has radiolar eyes and is Pseudopotamilla saxicava (de Quatrefages, 1866), a species previously synonymized with Pseudopotamilla reniformis (Müller, 1771) by McIntosh (1923: 233). At the beginning of 1990’s Knight-Jones examined type materials of Pseudopotamilla saxicava and noted that it had unique characters, making this species distinguishable from Pseudopotamilla reniformis. As shown in her poster presented during the 8th International Polychaete Conference in Madrid, Dr. Knight-Jones was working on the reestablishment of Pseudopotamilla saxicava. Unfortunately, she died before a full revision of the genus Pseudopotamilla had been completed.

## Supplementary Material

XML Treatment for Parasabella
